# Characterization of an Antioxidant and Antimicrobial Extract from Cool Climate, White Grape Marc

**DOI:** 10.3390/antiox8070232

**Published:** 2019-07-20

**Authors:** Kenneth J. Olejar, Arianna Ricci, Simon Swift, Zoran Zujovic, Keith C. Gordon, Bruno Fedrizzi, Andrea Versari, Paul A. Kilmartin

**Affiliations:** 1 School of Chemical Sciences, The University of Auckland, Private Bag 92019, Auckland 1142, New Zealand; 2 Department of Wine, Food and Molecular Bioscience, Lincoln University, P.O. Box 85084, Lincoln 7647, New Zealand; 3 Department of Agricultural and Food Sciences (DISTAL), University of Bologna, Piazza Goidanich 60, 47521 Cesena (FC), Italy; 4 Department of Molecular Medicine and Pathology, The University of Auckland, Private Bag 92019, Auckland 1142, New Zealand; 5 NMR Centre, The University of Auckland, Private Bag 92019, Auckland 1142, New Zealand; 6 Dodd Walls Centre, Chemistry Department, The University of Otago, P.O. Box 56, Dunedin 9054, New Zealand

**Keywords:** antioxidant activity, antimicrobial activity, grape tannin extract, phenolic compounds, waste valorization

## Abstract

Valorization of agricultural waste has become increasingly important. Wastes generated by wineries are high in phenolic compounds with antioxidant and antibacterial properties, which contribute to phytotoxicity, making their immediate use for agricultural means limited. Utilizing a water-based extraction method, the phenolic compounds from winery waste were extracted and purified. The resulting extract was characterized for phenolic composition using high-pressure liquid chromatography-ultraviolet/visible and electrochemical detectors (HPLC-UV/Vis, ECD) for monomers, and spectral assessment of the tannins present using attenuated total reflectance- Fourier transform infrared (ATR-FTIR), FT-Raman, and solid-state nuclear magnetic resonance (SSNMR) spectroscopies. The extract’s antioxidant activity was assessed by the scavenging of the 2,2-diphenyl-1–picrylhydrazyl (DPPH) radical and Folin-Ciocalteu total phenolic assay, and was found to be as effective as a commercially obtained grape extract. The extract’s antimicrobial efficacy was tested for minimum bactericidal concentration using *Candida albicans, Escherichia coli* 25922, and *Staphylococcus aureus* 6538, which resulted in greater efficacy against gram-positive bacteria as shown over gram-negative bacteria, which can be linked to both monomeric and tannin polyphenols, which have multiple modes of bactericidal action.

## 1. Introduction

Agro-wastes resulting from the production and processing of agricultural material create a unique set of challenges and opportunities. In the wine industry, agro-waste is generated in large quantities during harvest production, typically lasting 2 to 3 months of the year. These wastes contain many plant polyphenols, which are free radical scavengers and possess antimicrobial activity [1,2].

Grape phenolic compounds have multiple structures, ranging from simple mono-substituted phenolic acids to multi-substituted ring systems and tannins [3]. These structures can also form more complex combinations, resulting in dimers, trimers, and polymeric chains of the original monomer [4,5]. The most recognized group of monomers exhibiting these attributes is the flavanols, which have a C6–C3–C6 carbon bond structure [3]. Flavanols form polymeric chains through C4–C8 or C4–C6 bonding of the C and A rings for Type B proanthocyanidins [5]. These polymeric units are reactive towards proteins, with increased protein affinity when the polymer remains flexible by having an increased ratio of C4–C8 to C4–C6 bonds [6].

Grapes produce phenolic compounds in response to plant stressors. These stressors range from drought conditions to mold infection and UV radiation. Production of the phenolic compounds occurs within the cells of the grape, leaves, and the woody portions of the vine [7,8]. Within the grape, the seeds are particularly high in monomeric- and polymeric-based flavanols, while the skin contains mostly flavanols and flavonols [9]. During wine production, these compounds are extracted during maceration, although not all are removed. In particular, white wines typically do not undergo maceration, and grape skin contact with the juice is minimal prior to fermentation. While alcohol aids in the extraction process with red grapes, white grape marc has larger amounts of phenolics available.

Studies on phenolics have linked individual compounds and combinations of compounds to antioxidant, antimicrobial, and antiviral activities in both in vitro and in vivo studies [10,11,12,13]. Villano et al. [14] performed studies to calculate an antioxidant efficiency coefficient based on the ability of individual phenolics to scavenge the 2,2-diphenyl-1–picrylhydrazyl (DPPH) free radical, and established reaction rate constants. This coefficient allows for a better understanding of the antioxidant activity possessed by a collection of these compounds, such as in a grape extract.

Several methods have been developed and used in industry for the removal of phenolic compounds and other components from winery wastes, such as tartaric acid, colorants, and tannins. These methods have focused on extracting large quantities of phenolics by utilizing solvents, not all of which are approved for use in contact with food, or which involve expensive equipment, i.e., subcritical or supercritical fluids [15,16]. To make the extraction of phenolics possible as an industry option, the costs of extraction and the equipment employed must be less than the receivable profits. In addition, the ability to utilize the extract in applications associated with foods and/or personal hygiene expands the available market. In order to minimize the costs associated with solvents utilized and subsequent disposal/recovery, minimize the costs of equipment, and reduce procedural steps prior the purification process, water was selected as the extraction solvent [17].

The aim of this work was to evaluate a grape marc extract established from a water-based extraction method in order to minimize extraction costs and increase the value of the waste. Characterization of the extract was undertaken to better understand the compounds present that contribute to the antioxidant and antimicrobial activities. The extract make-up was determined through Fourier-transform infrared FTIR and FT-Raman spectroscopies for the presence of polymerization of monomeric phenolic compounds, high-pressure liquid chromatography with an ultraviolet/visible detector (HPLC-UV/Vis) and electrochemical detector (ECD) for monomeric phenolic compound quantification, and solid-state nuclear magnetic resonance (SSNMR) for tannin presence. The extract was also measured for antioxidant activity by the Folin-Ciocalteu total phenolic assay and DPPH radical scavenging assay. Antimicrobial testing to establish the minimum bactericidal activity was performed using *Candida albicans* (*C. albicans*)*, Escherichia coli* 25922 (*E. coli*)*,* and *Staphylococcus aureus 6538* (*S. aureus*). This work was undertaken in order to explore the potential for utilizing the extract as a natural alternative to synthetic antioxidants and antimicrobials.

## 2. Materials and Methods

### 2.1. Phenolic Compound Extraction

Utilizing the grape marc waste stream of a winery, bulk waste from the 2013 vintage was obtained, containing white grape skins, seeds, and residual leaf and stem materials. The marc was thoroughly mixed to produce a homogenous sample prior to division into two replicates for extraction. The grape marc was stored at −20 °C for less than 1 month prior to processing. In order to reflect the actual conditions during processing, the waste consisted of a mixture of varieties, Sauvignon blanc and Chardonnay, that were being processed, but was predominantly Sauvignon blanc.

The white grape marc, 4.5 kg wet weight, was ground using a food processor with equal parts by volume of purified water (Barnsted Nanopure, 18 Ω water system, Thermo Scientific, Waltham, MA). The resulting slurry was then extracted with 18 Ω water in a final volume of approximately 20 L for 24 h. The extract was then filtered using a sieve to remove the marc from the filtrate. The marc was then extracted once more with 20 L of water.

The filtrates were then combined and passed through a column (2.5 × 60 cm) packed with Amberlite FPX-66 resin, purchased from Dow Chemicals Ltd. (Auckland, New Zealand). The column was washed with three volumes of water. The phenolic compounds were released by passing two volumes of ethanol (Scharlau, Sentmenat, Spain) through the column. The ethanol fraction was collected and concentrated using a Roto-Vap (Savant, Thermo Fisher Scientific, Waltham, MA, USA). The concentrate was then dehydrated in a Speed-Vap (Thermo Savant, Waltham, MA, USA).

A commercially obtained extract of grape tannin derived from grape seeds was obtained from Tarac Technologies PTY LTD (Nuriootpa, SA, Australia). The commercial extract was used for comparison analysis in the Folin-Ciocalteu total phenolic assay, DPPH radical scavenging assay, antimicrobial analysis, and Rebelein residual sugar analysis.

### 2.2. Antioxidant Activity (Folin-Ciocalteu Total Phenolic Content)

Total phenolic content was established by the Folin-Ciocalteu assay, as described by Bajčan et al. [18]. The obtained extract was weighed and dissolved in 50% ethanol (Scharlau) solution at a concentration of 1 mg/mL; standards were also prepared in a 50% ethanol solution. In a 50 mL volumetric flask, 1.0 mL of extract solution or gallic acid standard (Sigma-Aldrich, St. Louis, MO, USA), 5.0 mL of distilled water, and 0.25 mL of Folin-Ciocalteu reagent (Sigma-Aldrich) were combined and incubated at room temperature for 3 min. Following incubation, 3.0 mL of 20% w/v sodium carbonate (Sigma-Aldrich) was added to each flask and brought to volume with distilled water prior to being placed at room temperature, where they were shielded from light for 90 min. The absorbance of the samples was then measured at 765 nm. The assay was performed in triplicate and results were reported in gallic acid equivalents (GAE)/g of extract.

### 2.3. Antioxidant Activity (DPPH Radical Scavenging)

The 2,2-diphenyl-1–picrylhydrazyl (DPPH) (Sigma-Aldrich) radical scavenging assay was performed using a spectrophotometer at 515 nm to determine the antioxidant activity of each extract, utilizing a modified method outlined in Villano et al. [14]. Briefly, extracts were dissolved in 80% methanol (Scharlau) at a concentration of 1 mg/mL. DPPH was dissolved in 80% methanol at a concentration of 63.4 μmol/L. To 20 mL of the DPPH solution, 100 μL of the extract solution was added and incubated at room temperature for 24 h, protected from light. The 63.4 µmol/L DPPH solution was serially diluted to make a standard curve of DPPH concentration, which was measured on the same day as preparation. The 63.4 µmol/L DPPH solution was incubated with the samples to serve as a blank. The assay was performed in triplicate, and the results reported as percent DPPH scavenged and the 50% inactivation of DPPH as µmol DPPH/mg of extract.

### 2.4. HPLC for Monomeric Phenolic Determination

Phenolic compounds were determined by HPLC-UV/Vis and ECD, using the method described by Olejar et al. [19]. Compound identification was confirmed by retention time of standards and UV spectra, while the quantification of compounds was done using the ECD, due to its increased sensitivity. Individual phenolic compounds are reported in μg/mg of extract. The extract samples were prepared in duplicate by dissolving 1 mg of extract in 1 mL of a 10% ethanol solution to mirror the monomeric standards preparation. Extract and standard solutions were filtered through 0.2 μm polytetrafluoroethylene (PTFE) syringe filter (Pall Corp., Hamilton, New Zealand), and 20 μL of the filtrate was injected into an Agilent 1100 HPLC with a UV/Vis detector (Santa Clara, CA, USA) and an ESA Coulochem III electrochemical detector (Waltham, MA, USA). Chromatography occurred at 1.0 mL/min over 30 min at 40 °C on a 3.0 × 100 mm, 3 μm, Supelco Ascentis RP-amide column (St. Louis, MO, USA). Analyte separation was performed using a gradient elution of mobile phase A: 30 mM phosphate buffer at pH 2.6, and mobile phase B: a mix (30:10:60) of 100 mM phosphate buffer, methanol, and acetonitrile at pH 2.6. The gradient was 0–10 min 12% B, 10–15 min 30% B, 15–17.5 min 55% B, 17.5–21 min 55% B, 21–23 min 100% B, and 23–25 min 0% B. Detection of analytes was undertaken at 280, 305, 320, and 365 nm for UV, as well as at 450 and 750 mV using a guard cell set to 1000 mV for the for ECD. Standard solutions were made at an initial concentration of 1 mg of standard/mL of 10% ethanol. Serial dilutions were then performed to generate a standard curve. The standards utilized in this analysis were: gallic acid, syringic acid, *p*-coumaric acid, caffeic acid, ferulic acid, resveratrol, (+)-catechin, (−)-epicatechin, epicatechin gallate, epigallocatechin gallate, kaempferol, quercetin, and rutin.

### 2.5. Spectroscopic Analysis

The attenuated total reflectance (ATR) spectra were registered using a diamond ATR Smart Orbit™ accessory (from Thermo Optec) with a bouncing refractive infrared beam at 45° angle of incidence, using a deuterated triglycine sulfate (DTGS) detector with a KBr window, operating in the mid-IR. The spectra were obtained by placing the phenolic extract on the macro ATR crystal and applying pressure to optimize contact, and were scanned over the range of 4000–650 cm^−1^ with resolution 4 cm^−1^ and averaged over 128 scans. Standard software (Omnic ESP, version 7.2, Waltham, USA) was used for data acquisition and analysis.

FT-Raman spectra were collected on the phenolic extract using a Bruker MultiRAM instrument (Bruker, Ettlingen, Germany). A liquid nitrogen–cooled germanium diode detector was used, controlled by the Bruker OPUS v6.0 software. The excitation source was a Nd:YAG 1064 nm laser, operating in the near infrared (NIR) region. In order to avoid sample burning, the laser was defocused, resulting in a 2 mm laser spot size with 500 mW power applied. A total 128 scans were averaged over every acquisition, with a spectral resolution of 4 cm^−1^ in the wavenumber range of 3500–0 cm^−1^. Bruker OPUS v6.0 software was used for data acquisition (Bruker, Ettlingen, Germany), while spectral corrections (baseline correction and smoothing: 9 points) were performed with Omnic ESP, version 7.2 (Thermo Scientific, Waltham, MA, USA).

### 2.6. Solid-State Nuclear Magnetic Resonance (SSNMR)

The solid-state NMR experiments were performed using a Bruker Avance 300 spectrometer operating at 300.13 MHz proton frequency. A multinuclear, double-tuned Bruker probe with 7 mm zirconia rotors, retained with Kel-F end-caps was used. The ^13^C CP/MAS spectra were recorded using a 4.2 µs 90° proton pulse, with a recycle delay of 1.5 s and a spin rate of 7.0 and 4.0 kHz to check the sidebands. A contact time of 1 ms was used for the ^13^C CP/MAS experiments. ^1^H decoupling was obtained via the continuous wave approach. The number of scans was 2600. All spectra were referenced (externally) to adamantane (38.48 ppm) and were processed using TopSpin^TM^ NMR Software (version 2.1, Bruker, Alexandria, NSW, Australia).

### 2.7. Antimicrobial Activity

Extracts were tested for minimum bactericidal concentration (MBC) against *Escherichia coli* ATCC25922, *Staphylococcus aureus* ATCC6538, and *Candida albicans* ATCC1212 (Genetic Stock Center, Yale University, New Haven, USA) [20,21]. Briefly, 2% (*w*/*v*) concentrations of the extracts in tryptic soy broth (TSB) (Hach Pacific, Penrose, Auckland, New Zealand) were subjected to a serial dilution, where each dilution is half the concentration of the prior, in 96 well, flat-bottomed polystyrene microtiter plates. To these, approximately 1 × 10^6^ CFU/mL was added and incubated at 37 °C at 200 RPM for 24 h. From each well, 20 μL was removed and drop plated on a TSB agar plate (Hach Pacific, Penrose, Auckland, New Zealand) and allowed to dry. Following incubation at 37 °C for 24 h, the appearance of colonies was noted to establish the MBC. Bactericidal concentration was established as a reduction of 1 × 10^3^ CFU/mL. Extracts were assayed in triplicate, three times. Averages are reported as percent of extract required to cause inhibition.

### 2.8. Rebelein Residual Sugar

Residual sugars in the extracts were determined by the Rebelein method [22]. Briefly, 10 mg of extract was dissolved in 5 mL of water for a final solution concentration of 2 mg extract/mL of water. Into a 200 mL flask, 10 mL of a 0.168 M copper sulfate (Fisher Scientific, Hampton, NH, USA) in 0.01 N sulfuric acid solution (BDH Chemicals, Ltd., Randor, USA), 5 mL of 0.886 M Rochelle salt (Ajax Finechem, Taren Point, Australia) in 2 M sodium hydroxide (Fisher Scientific) and 2 mL of sample were combined. The mixture was heated rapidly until steam was derived and maintained at temperature for 1.5 min, after which it was rapidly cooled in an ice bath. To the cooled mixture, 10 mL of 1.81 M potassium iodide (May & Baker PTY LTD, Sydney, Australia) in 0.1 N sodium hydroxide, 10 mL 16% sulfuric acid (Fisher Scientific), and 10 mL of a 1% starch indicator (Fisher Scientific) in 0.120 M potassium iodide in 0.01 N sodium hydroxide were combined. Titration occurred with 0.056 M sodium thiosulfate (Sigma-Aldrich) in 0.05 N sodium hydroxide. Analysis was performed in triplicate, and averages reported as mg of sugar/g of extract.

### 2.9. Statistical Analysis

Basic data analysis was performed using Microsoft Excel 2011 for Mac (version 14.4.7, Microsoft Corporation, Redmond, WA, USA). Statistical T-test analysis was performed on the antioxidant activity (Folin-Ciocateu and DPPH), antimicrobial, and Rebelein residual sugar assays using JMP for Mac (version 11.2.1, SAS Institutes Inc., Cary, NC, USA).

## 3. Results and Discussion

### 3.1. Extraction

Extraction with water and subsequent Amberlite purification yielded 1.3% (*w*/*w*) of extract. Although this value is in the range provided by other publications [15,23], the obtained value is considered low due to two factors: the extraction solvent being water and the purification step. Water has been shown not to be as effective as organic solvents when used alone in the extraction of phenolics from grape marc [24], however, its low-cost, non-toxic nature, and minimization of processing steps for purification make it an ideal solvent. Spigno et al. [16] evaluated the effects of time, temperature, and solvent on the extraction of phenolics from grape marc, finding that elevated temperatures and a mixture of solvents increased the extract yield. The current extraction at room temperature may be modified to maximize the extract obtained from the aqueous process by increasing the water temperature, however, the increase in overall extract gains must be reconciled with the economic cost associated with increasing the water temperature. Additionally, the purification step with Amberlite, which binds phenolic compounds, can permit some phenolics to pass through the column unbound when saturated or sterically hindered. The decrease in phenolics caused by purification is outweighed by the benefits of lower extraneous materials, such as carbohydrates (sugar), lipids, and other non-phenolic-based materials.

### 3.2. Monomeric Phenolic Compounds Determined by HPLC-UV/Vis-ECD

The phenolic profile of the extract is given in Table 1. The phenolic profile of the extract shows high levels of flavan-3-ols (47.1 ± 1.9 μg/mg of extract). Flavan-3-ols are considered highly reactive as a result of their ability to form intermolecular bonds between molecules and with gallic acid, as seen by Yilmaz, Toledo [25], where the superior antioxidant activity of grape seeds could be attributed to the formation of these compounds. Galloylation and polymerization of flavanols has been observed to increase the antioxidant activity of the molecule [26]. Monomeric phenolic acids made up 16.9 ± 0.4 μg/mg of the extract. The detected monomeric phenolic profile was only a portion, 7% by weight, of the active compounds present in the extract, as there were dimeric, trimeric, oligomeric, and polymeric compounds, as well as impurities, constituting the remaining 93%. Confirmation of the presence of these compounds came from the ATR-FTIR, FT-Raman, and solid-state NMR studies.

### 3.3. Spectroscopic Techniques

#### 3.3.1. ATR-Fourier Transform Infrared Spectroscopy

**Figure 1:**
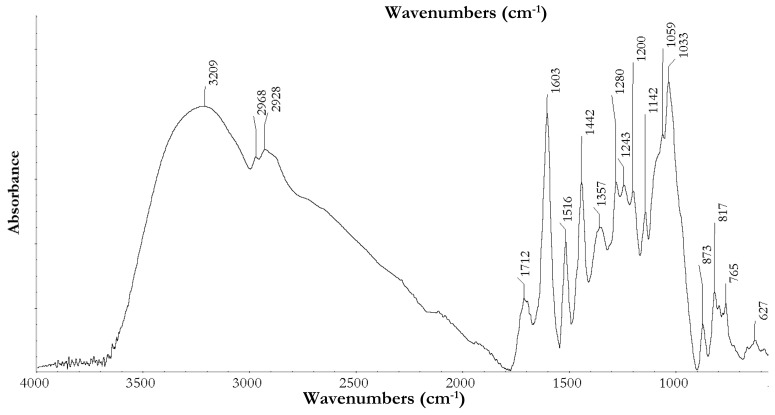
Extract spectral analysis performed using Attenuated Total Reflection Fourier-transform Infrared (ATR-FTIR) spectroscopy.

ATR-FTIR analysis of the extract indicated the presence of a mix of hydrolysable and condensed tannins (Figure 1). Hydrolysable tannins, found in grape skins and seeds [27,28], were identified by the broad peak at 1712 cm^−1^, attributable to carbonyl stretch, peaks at 1200 cm^−1^ and 1033 cm^−1^ of the C–O asymmetric and symmetrical stretching, and peaks at 873 cm^−1^, 765 cm^−1^, and 1088–1082 cm^−1^, which are obscured by the intensity and broadening of the peak at 1030 cm^−1^. Condensed tannins were identified through shoulders on the broad peaks at 1162–1155 cm^−1^, 1116–1110 cm^−1^, 974 cm^−1^, and 844–842 cm^−1^. Additionally, the peak at 1516 cm^−1^, which indicates the presence of non-gallate procyanidins, as a doublet, which is commonly associated with flavanol gallate skeletal stretch of the *o*-distributed aromatic B-ring, was not observed. Furthermore, there was a C–C–OH deformation of a flavanol heterocyclic ring, as indicated by the doublet at 840–795 cm^−1^, which also indicates a mixture of tannins in the *cis* and *trans* configuration, with *trans* being predominant, as confirmed by the peak at 1603 cm^−1^ [29].

The peak at 1603 cm^−1^ is the C=C–O deformation of the heterocyclic C-ring in the dominant planar *trans* form. The *cis* configuration is less sterically constrained, and therefore produces a band shift to a higher frequency. The large, broad peak at 3660–3000 cm^−1^ is indicative of an OH stretch of a benzene nucleus and methyl tannin groups, with a peak at 3400–3200 cm^−1^ for the OH stretch of tannins. Peaks at 2968 cm^−1^ and 2928 cm^−1^ are due to symmetrical and asymmetrical stretches of CH_2_ groups [29].

#### 3.3.2. Fourier Transform Raman Spectroscopy

**Figure 2:**
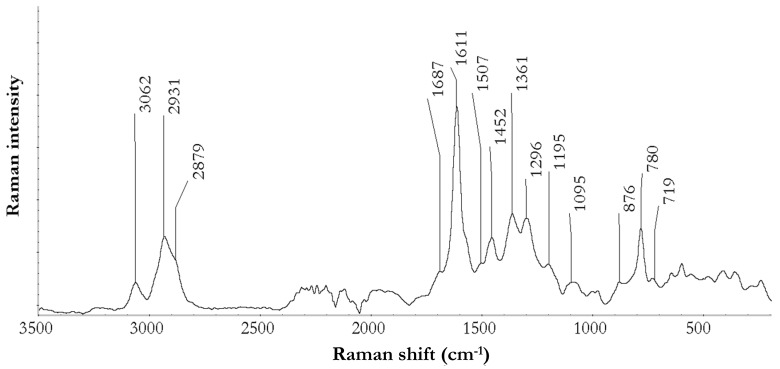
Fourier-transform Raman (FT-Raman) spectra of purified grape marc extract.

FT-Raman exhibited a number of diagnostic peaks and a generic low intensity of signals, but it provided useful information about the composition of the extract (Figure 2). Several weak signals occurring in the region less than 600 cm^−1^ were attributable to a combination of C–C and C–O vibrational motions, which can be due to both the heterocyclic C-ring of flavonoid-like structures, and C–O linkages of hydroxyl groups in hydrolysable compounds [30]. The peak at 719 cm^−1^ was specifically assigned to the –CH out-of-plane vibrations of the *o*-substituted aromatic ring of flavonoid compounds, coupled to the 1507 cm^−1^ weak band due to the in-plane deformation of the B phenyl ring [31]. The 780 cm^−1^ peak was probably related to CH out-of-plane deformation of aromatic substituted rings, but the exact attribution is controversial. The 1361 cm^−1^ peak was reported as a combination band between C–C stretching of aromatic rings and ring deformation vibrations, and the expected related vibration around 1331 cm^−1^ was shifted to lower frequencies for this sample, at 1296 cm^−1^ [32]. Authors attributed this shift to the occurrence of the polymerization process, which decreases the ability of the aromatic quadrant to undergo vibrational deformations, and it has been taken as evidence of the polymerization of phenolic compounds. Several authors agree that a strong peak occurring at around 1611 cm^−1^ is diagnostic of the presence of phenolic compounds, and it is related to C–CH quadrant stretching modes [30,33]. A further signal present as a shoulder around 1687 cm^−1^ was related to combination vibrations, where C=C and C=O moieties are involved; [30] the latter is attributable to hydrolysable structures. A noisy spectrum between 1700 and 2000 cm^−1^ made the recognition of bands occurring for C=O carbonyl and carbonyl ester groups difficult. The peak at 1452 cm^−1^, coupled with a broad signal centered around 2879 cm^−1^, could be attributed to the presence of methyl esters; these were assigned to CH bending in the former case, and the stretching of aliphatic CH in the latter [30]. A further consideration about the extent of polymerization for this extract involves the aliphatic CH stretching (broad band) and the C–O stretching occurring at 1095 cm^−1^ (weak signal); these peaks are weakened in the case of polymeric structures, as was observed for lignin, [34] while there is a relative increase in signal related to aromatic structures, as observed here at 2931 and 3062 cm^−1^ (CH stretching of aromatic rings), and 1611 cm^−1^.

### 3.4. Solid-State Nuclear Magnetic Resonance

**Figure 3:**
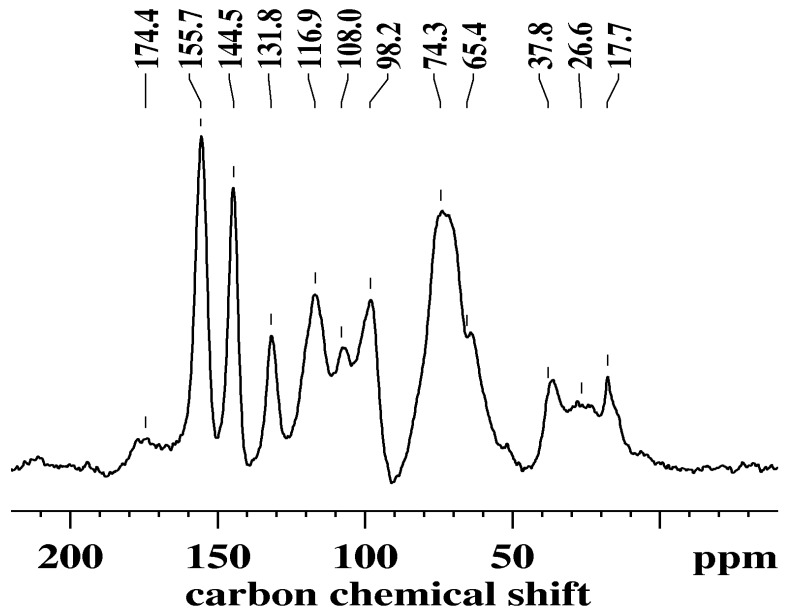
Carbon-13 solid-state nuclear magnetic resonance (SSNMR) spectra of grape marc extract with the main peaks labeled.

The SSNMR analysis (Figure 3) supports the FTIR interpretation of the extract being rich in tannins. The peak at 108.0 ppm can be attributed to the interflavanoid bonds at C4–C8 [35,36]. The lack of a peak around 95 ppm, which was previously attributed to the C4–C6 interflavanoid bond, [36] combined with the presence of the peak at 108.0 ppm, suggests that the structural units are mostly linked to the C4–C8 bond, which represents the prevalent structure within the tannin formation in the extract.

The low intensity, broad peak centered at 174.4 ppm can be associated with the gallic acid residue linked to the C3 of the heterocyclic ring of the flavonoid, more specifically, the C=O of the gallic residue linked to a catechin or epicatechin [36].

Peaks relating to OH moieties connected to the A-ring of flavonoids were seen at 155.7 ppm (C5, C7), and for the B-ring, 144.5 ppm (C3’, C4´) and 116.9 ppm (C5´) [36]. The peak at 131.8 ppm is indicative of the bond at C1´ connecting the B and C-rings [37]. The OH moiety on the C-ring at C3 was represented as a shoulder at 65.4 ppm [37]. This peak was very broad in nature and therefore covered the C2 bands.

Free C in the flavonoids was seen as peaks at 98.2 ppm (C6, C8, C10) and a shoulder at approximately 20 ppm (C4) [26,38]. The peak at 37.8 ppm was attributed to C4 involved in inter-flavanoid bonding [39]. The larger intensity of this peak compared to the one at 20 ppm suggests that the extract was highly polymeric in nature, further confirming the assignment of the peak at 108.0 ppm.

### 3.5. Antioxidant Activity

It is well known that antioxidant activity is directly related to the phenolic content of an extract. As outlined by Villano et al. [14], individual phenolic compounds have differing reaction rates and abilities to scavenge radicals. These traits, combined with the individual phenolic concentrations, result in the observed antioxidant activity. Antioxidant activity was measured through the use of DPPH radical scavenging and the Folin-Ciocalteu method, commonly referred to as the total phenolic content, however, the assay reacts with any reducing agent, thereby making it an assessment of antioxidant activity [40].

The antioxidant activity (DPPH radical scavenging) and total phenolic contents (Folin-Ciocalteu method) of the extract and a commercially available extract were compared and are shown in Table 2. The total phenolic content was 255 ± 3 mg GAE/g for the obtained extract and 258 ± 4 mg GAE/g for the commercial extract, respectively. These results are within the range of total phenolics found by Apostolou et al. [41] of 167 and 444 mg GAE/g for grape pomace and grape seed extracts, and Sagdic et al. [42] in grape pomace, 75 to 288 mg GAE/g extract, according to the grape cultivar.

The values for the DPPH radical scavenging of the two extracts were: extract 1.15 ± 0.06 μmol DPPH/mg and commercial 1.01 ± 0.06 μmol DPPH/mg, or 83.9 ± 0.8% and 81.5 ± 0.3% scavenging, respectively, for this test procedure. Similar results were found for the DPPH radical scavenging in a previous study involving Pinot meunier, 0.0723 to 1.18 μmol DPPH/mg extract, and Pinot noir, 3.9 to 6.6 μmol DPPH/mg extract, depending on the organic solvent type utilized at 50% DPPH inactivation [43]. For comparison, converting the results of the current study to 50% inactivation values, 0.685 and 0.620 μmol DPPH/mg of extract was calculated for the obtained and commercial extracts, respectively, which are values within the previously reported ranges. This result also demonstrates that the water-only extraction process can produce an end product with similar scavenging activity to extracts obtained from organic solvent extraction.

### 3.6. Antimicrobial Activity

Table 2 shows the minimum bactericidal concentration (MBC) of the extract, which was found to be 0.125% (*w*/*v*) extract against *S. aureus*, and 2.0% (*w*/*v*) extract against *E. coli*. In contrast, the commercially obtained extract had a MBC of 0.125% (*w*/*v*) extract against *S. aureus* and was not effective against *E. coli* at the tested concentrations. It can be seen that the extracts were more effective against *S. aureus*, a gram-positive bacteria. Neither extract displayed effectiveness against *C. albicans* at the tested concentrations. This activity profile is supported in several other studies utilizing gram-positive bacteria [43,44]. Tesaki et al. [45], investigating the activity associated with grapes, were further able to identify gallic acid as the compound effective against gram-positive bacteria. The current study suggests that there is efficacy for use of the extract at concentrations above 0.125% (*w*/*v*) extract for bactericidal application against gram-positive bacteria, and suggests efficacy for gram-negative bacteria at the higher concentration of 2% (*w*/*v*) extract. Further testing to expand the spectrum of bacteria affected by exposure to the extract is needed. However, the literature supports the use of grape extract against many types of bacteria [44,46].

### 3.7. Extract Residual Sugar

Comparison of residual sugar contents in the two extracts shows a three-fold decrease in sugar content of the obtained extract over the commercial one (70 ± 33 and 233 ± 66 mg/g, respectively). The purification process of the aqueous extract lowered the sugar content. However, the FTIR and FT-Raman spectra suggest that there were hydrolysable tannins in the extract, which may account for some of the sugar detected, as sugar molecules are attached to the phenolic moieties in this case.

## 4. Conclusions

This study shows the effectiveness of a water-based extraction method for obtaining a viable polyphenol extract, which has been produced using methodologies that minimize environmental impact and cost of extraction. Characterization of the extract shows the proposed aqueous process produces a product with minimal residual sugar that may potentially limit its applications, such as where heating of the extract is required. It has been demonstrated that a useful crude extract can be obtained, which needs no further purification or constituent isolation for antimicrobial or antioxidant activity. The antioxidant and antimicrobial activity against gram-positive bacteria at low concentrations showed the potential for applications of aqueous extracted grape tannins, including as a packaging material additive to protect contents against bacterial and oxidative spoilage. The combination of monomeric and tannic phenolic compounds provides a range of compounds for protection, as the monomeric compounds have a decreased steric hindrance, while the tannins provide multiple active sites, as well as increased scavenging ability [47]. This methodology valorizes the waste stream and establishes the groundwork for further research into conditions and technologies aimed at maximizing extract yield using aqueous methods and its potential applications based on the phenolic make-up needed.

## Figures and Tables

**Table 1:** Phenolic profile of the monomers in the extract, expressed as μg per mg of extract, unless otherwise noted.

Phenolic Compound	μg/mg of Extract
Phenolic acids	
Gallic acid	2.63 ± 0.03
Ferulic acid	6.59 ± 0.13
*p*-Coumaric acid	2.01 ± 0.11
Caffeic acid	5.63 ± 0.32
Sum of phenolic acids	16.9 ± 0.4
Flavan-3-ols	
(+)-Catechin	7.01 ± 0.02
(-)-Epicatechin	16.8 ± 1.1
Epicatechin gallate *^†^*	15.4 ± 1.5
Epigallocatechin gallate	7.89 ± 0.40
Sum of flavan-3-ols	47.1 ± 1.9
Flavonols	
Quercetin glycoside ^‡^	7.59 ± 0.08
Sum of monomeric phenolics	c. 70 (i.e., 7% by weight)

*^†^* Expressed in (+)-caffeic acid equivalent, ^‡^ Reported in rutin equivalent.

**Table 2:** Results of antioxidant and antimicrobial testing of experimental and commercial extracts.

**Antioxidant Activity**	**Experimental Extract**	**Commercial Extract**
Folin-Ciocalteu (mg GAE/g extract)	255 ± 3	258 ± 4
DPPH (µmol DPPH/mg extract)	1.15 ± 0.06	1.01 ± 0.06
DPPH (% radical scavenged)	83.9 ± 0.8	81.5 ± 0.3
**Antimicrobial Activity (MBC)**	**Experimental Extract**	**Commercial Extract**
*Staphylococcus aureus* (% *_w_*_/*v*_)	0.125	0.125
*Escherichia coli* (% *_w_*_/*v*_)	2.0	N.E.
*Candida albicans* (% *_w_*_/*v*_)	N.E.	N.E.

GAE—Gallic acid equivalents, DPPH—2, 2-diphenyl-1-picrylhydrazyl, MBC—Minimum bactericidal concentration, N.E.—No effect detected.
